# Antimicrobial-specific response from resistance gene carriers studied in a natural, highly diverse microbiome

**DOI:** 10.1186/s40168-020-00982-y

**Published:** 2021-01-27

**Authors:** Wisnu Adi Wicaksono, Peter Kusstatscher, Sabine Erschen, Tamara Reisenhofer-Graber, Martin Grube, Tomislav Cernava, Gabriele Berg

**Affiliations:** 1 Institute of Environmental Biotechnology, Graz University of Technology, Graz, Austria; 2 Institute of Biology, University of Graz, Graz, Austria

**Keywords:** Lichen microbiota, *Peltigera polydactylon*, Antimicrobial resistance, Metagenomic mining, Genome recovery

## Abstract

**Background:**

Antimicrobial resistance (AMR) is a major threat to public health. Microorganisms equipped with AMR genes are suggested to have partially emerged from natural habitats; however, this hypothesis remains inconclusive so far. To understand the consequences of the introduction of exogenic antimicrobials into natural environments, we exposed lichen thalli of *Peltigera polydactylon*, which represent defined, highly diverse miniature ecosystems, to clinical (colistin, tetracycline), and non-clinical (glyphosate, alkylpyrazine) antimicrobials. We studied microbiome responses by analysing DNA- and RNA-based amplicon libraries and metagenomic datasets.

**Results:**

The analyzed samples consisted of the thallus-forming fungus that is associated with cyanobacteria as well as other diverse and abundant bacterial communities (up to 10^8^ 16S rRNA gene copies ng^-1^ DNA) dominated by *Alphaproteobacteria* and *Bacteroidetes*. Moreover, the natural resistome of this meta-community encompassed 728 AMR genes spanning 30 antimicrobial classes. Following 10 days of exposure to the selected antimicrobials at four different concentrations (full therapeutic dosage and a gradient of sub-therapeutic dosages), we observed statistically significant, antimicrobial-specific shifts in the structure and function but not in bacterial abundances within the microbiota. We observed a relatively lower response after the exposure to the non-clinical compared to the clinical antimicrobial compounds. Furthermore, we observed specific bacterial responders, e.g., *Pseudomonas* and *Burkholderia* to clinical antimicrobials. Interestingly, the main positive responders naturally occur in low proportions in the lichen holobiont. Moreover, metagenomic recovery of the responders’ genomes suggested that they are all naturally equipped with specific genetic repertoires that allow them to thrive and bloom when exposed to antimicrobials. Of the responders, *Sphingomonas*, *Pseudomonas*, and *Methylobacterium* showed the highest potential.

**Conclusions:**

Antimicrobial exposure resulted in a microbial dysbiosis due to a bloom of naturally low abundant taxa (positive responders) with specific AMR features. Overall, this study provides mechanistic insights into community-level responses of a native microbiota to antimicrobials and suggests novel strategies for AMR prediction and management.

**Video Abstract**

**Supplementary Information:**

The online version contains supplementary material available at 10.1186/s40168-020-00982-y.

## Introduction

Antimicrobial resistance (AMR) is an increasingly serious threat to global public health [1]. New resistance mechanisms are emerging and spreading globally, which reduces our means to treat common infectious diseases and therefore increasingly results in prolonged illness, disability, and death [1]. Current research suggests that the unexplored diversity of resistance mechanisms in environmental bacteria is a risk factor for the human population, and not only clinical pathogens that are equipped with AMR [2]. Natural environments are described as the origins and reservoirs of antimicrobial resistance genes (ARGs) [3]. Recent studies primarily focused on microbial communities and their ARGs in a wide range of human-influenced environments such as agricultural farmland, crop plants, food production systems, and wastewater treatment plants [4–7]. However, to fully understand the evolution, emergence and spread of antimicrobial resistance, it is crucial to also study natural systems that are not disturbed by anthropogenic factors.

Microbial diversity within natural microhabitats is an important bioindicator of changes in ecosystem function due to disturbances, such as exposure to pollutants, agricultural practices, climate change [8], and exposure to antimicrobials (reviewed in [9]). Generally, the effects of antimicrobials on single microorganisms or small consortia are well-known; however, understanding the consequences of antimicrobial exposure in complex, host-associated microbiomes is a critical area where more research is required. It is important because the changes induced by antimicrobial exposure can have an immediate effect on host health [9]. Exposure to antimicrobial substances can severely impact microbial communities and often leads to selection and/or enrichment of ARGs [10, 11]. A recent study by Mahnert et al. [12] demonstrated that loss of microbial diversity, due to cleaning in confined environments such as intensive care units and cleanroom facilities, correlates with an increase of antimicrobial resistance features. Despite this growing body of research that links antimicrobial exposure to changes in microbial communities, the community response to antimicrobial exposure in native environments is not yet understood [13].

Appropriate models for native microbial communities as well as for antimicrobial substances are required to obtain mechanistic insights into the effects of antimicrobial exposure on natural microbiota. Synthetic microbial communities are often used to simulate natural systems; however, they are less complex and less diverse and have lower functional connectivity than natural microbiota. Lichens form spatially limited microbial ecosystems consisting of a fungus (mycobiont), eukaryotic algae and/or cyanobacteria (photobiont), and thousands of different bacterial species [14–16]. Lichen-associated bacteria carry unique functional properties adapted to the holobiont, such as the production of antimicrobial substances and resistance towards toxic compounds [14, 17, 18]. Although many lichens can persist under environmental extremes when they are dehydrated, they are generally vulnerable to slight changes in their microclimate [19], which substantially affects the fine-tuned symbiotic interplay [20]. We have selected lichens as promising systems for the exploration of complex, community-level responses of the microbiome to antimicrobial exposure due to their widespread use as models for classical symbioses as well as for bio-indication/monitoring approaches [21]. Lichen thalli were exposed for a defined time period to representative clinical antimicrobials with narrow (colistin) and broad-spectrum activity (tetracycline) [22, 23]. A bioactive alkylpyrazine was included to represent a novel, non-clinical antimicrobial [24] and glyphosate was included due to its wide but controversial use as herbicide with potential to affect the photobiont as well as non-target microorganisms [25, 26]. Despite its toxicity, colistin is regarded as a last-line antimicrobial for the treatment of Gram-negative multi-resistant bacteria in many regions [27]. Our hypothesis was that all antimicrobials will reduce bacterial richness, suppress naturally dominant taxa, and induce (visible) dysbiosis in the lichen symbiosis. Furthermore, due to different target spectra and modes of action of the four selected antimicrobial compounds, we expected varying responses of the bacterial communities and enrichment of taxa with specific resistance features.

For this purpose, we studied bacterial community responses in the ‘many-fruited pelt lichen’ *Peltigera polydactylon* (Neck.) Hoffm. during exposure to a full therapeutic dosage and a gradient of sub-therapeutic dosages of four antimicrobials (colistin, tetracycline, glyphosate, alkylpyrazine) by DNA- and RNA-based amplicon sequencing along with a metagenomic dataset analyses. Specifically, we addressed the following questions: (i) Is there a specific microbial shift induced by antimicrobial exposure? (ii) Which taxa respond to antimicrobial exposure? And (iii) which genetic reservoir allows positive responders to thrive under antimicrobial exposure? Overall, this study provides key insights on how antimicrobial exposure shapes microbial communities in their natural environments and provides insights into the potential consequences of modern antimicrobial overuse.

## Materials and methods

### Collection of lichen material and antimicrobial treatments

*Peltigera polydactylon* (Neck.) Hoffm. samples were collected in the proximate vicinity of a peri-urban area (Graz, Austria; 47° 06′ 45.6″ N, 15° 27′ 55.8″ E). The healthy lichen population is part of a natural forest landscape with no industrial zones in the close proximity. It is located on an elevation and thus not affected by potential run-off from surrounding farmland. The sampling location represents a relatively pristine environment that will be affected by progressing urbanization. All samples were visually examined to detect and remove macroscopic contaminants, such as adhering moss and plant detritus, with sterile tweezers. Following the initial pre-processing steps, lichen samples (0.5 g dry weight) were placed into sterile Petri dishes.

Four different antimicrobials including colistin sulphate (Sigma-Aldrich, USA), tetracycline (Merck, Germany), glyphosate (commercial herbicide Roundup® Alphée containing a glyphosate concentration of 7.20 g/l; Scotts Celaflor, Mainz, Germany), and an antimicrobial alkylpyrazine (5-isobutyl-2,3-dimethylpyrazine 97%, Sigma-Aldrich, USA) were used. As antimicrobial dosages could substantially impact the microbial community (reviewed in [8]), we selected dosages of the antimicrobials based on previous published studies that represented a full dosage (FD) and sub-therapeutic dosages (SD; 5-, 10-, and 20-fold dilution of FD) of each antimicrobial (in total 16 treatments; Table 1). Aqueous working solutions of each antimicrobial were prepared in sterile water as the solvent. Lichen samples were treated every 24 h with the antimicrobials over a period of 10 days by spraying the antimicrobial solutions (approximately 750 μL per treatment) onto the surface of the lichens. Negative controls were implemented where lichens were sprayed with sterile water. The lichen samples were kept at room temperature. During the experiment, the average relative humidity range was between 58 and 65% (average = 61.8%), whereas the average temperature was between 21 and 25 °C (average = 23.1 °C). Each treatment was performed in three biological replicates. After a 10-day incubation period, the samples were immediately transferred into a 15-ml reaction tube with RNAlater stabilization solution (Ambion, Life Technologies, Germany) and stored at − 80 °C until total nucleic acid extraction.

**Table 1:** Antimicrobial substances and their dosages used in this study

Antimicrobial	Full dosage	Sub-therapeutic dosages (*n* = 3)	Reference
Colistin*	300 mg/kg	60, 30, and 15 mg/kg	[28, 29]
Tetracycline*	1000 mg/kg	200, 100, and 50 mg/kg	[30]
Glyphosate	7.2 g/L	1.44, 0.7, and 0.36 g/L	[31]
Alkylpyrazine	0.66%	0.13, 0.07, and 0.03%	[32]

*The required amount of these antimicrobials was calculated based on fresh weight of lichen thalli

### Total nucleic acid extraction and cDNA synthesis

Total deoxyribonucleic acid (DNA) and ribonucleic acid (RNA), from approximately 100 mg of lichen sample, was extracted using the FastDNA™ SPIN Kit for Soil (MP Biomedicals, Germany) and TRIzol® Plus RNA Purification Kit (Ambion, Life Technologies), respectively, following the manufacturer’s instructions. To facilitate cell lysis, the samples were homogenized at room temperature using the FastPrep™ Lysing Matrix E and a FastPrep®-24 Instrument (MP Biomedicals, Germany) for 3 × 30 s at 6.0 m/s with 1 min in-between cooling on ice. The RNA and DNA quality and quantity were examined by using the NanoDrop™ 2000/2000c Spectrophotometer and Qubit dsDNA BR and Qubit RNA HS Assay Kit (Thermofischer Scientific), respectively. To remove genomic DNA, total RNA (100 ng) was treated with DNase I (Epicentre; Lucigen, USA) and subsequently used to synthetize complementary DNA (cDNA) using 5X All-In-One RT MasterMix (Applied Biological Materials, Richmond, BC, Canada) according to the manufacturer’s instructions. Prior further analysis, cDNA was diluted 10 times using nuclease-free water (Carl Roth, Germany).

### Quantification of bacteria in lichen samples

Quantitative real-time PCR (qPCR) based on SYBR Green fluorescence was performed to quantify the total and active bacterial density after antimicrobial treatment using the primer pair 515f–927r [33, 34]. In total, 51 DNA and 51 cDNA samples were analysed (three biological replicates of each treatment and concentration). The qPCR reaction mix contained 1 μL DNA/cDNA template, 5 μL KAPA SYBR® FAST qPCR Master Mix (2X) (KAPA Biosystem, USA), 1 μL 10 μM of each primer, and 3 μL ultrapure water. Fluorescence quantification was performed using the Rotor-Gene 6000 real-time rotary analyser (Corbett Research, Sydney, Australia) with initial denaturing at 95 °C for 10 min, followed by 40 cycles of denaturing at 95 °C for 30 s, annealing at 60 °C or 62 °C or 64 °C for 30 s, and extension at 72 °C for 30 s and a final melting curve. The Unibac-II fragment [33] was subjected to serial dilution (1:10) and run in two technical replicates to create qPCR standard. Negative and no-template controls were included in every run.

### Amplicon sequencing-based analyses of active and total bacterial communities

Extracted DNA and cDNA were subjected for amplicon polymerase chain reaction (PCR) to target the bacterial community. The primer set 515f/926r was used to amplify the V4-V5 region of bacterial 16S rRNA gene [35]. The primers were constructed to contain an overhang at the 5′ end that was used to attach barcodes and Illumina flow cell adapter sequences in the subsequent PCR as previously described in the protocols of the Earth Microbiome Project [36]. Two technical replicates were performed for each sample. The quality of the PCR products was checked visually by loading to 1% agarose gel electrophoresis and using ultra-violet light with Biorad Universal Hood II Gel Doc System (Biorad, USA). Barcoded PCR products were pooled in equimolar concentrations after purification using Wizard® SV Gel and PCR Clean-Up kit (Promega). The pooled library was sent to the Genewiz (Leipzig, Germany) and sequenced using Illumina MiSeq (v2 reaction kit) (2 × 300 bp paired-end).

### Bioinformatic analyses

Due to low quality of the reverse reads, we only used forward reads for the amplicon sequencing analysis. To confirm robustness of the conducted data analysis, we compared forward and paired-end read datasets and observed a congruent result with both analysis strategies (Table S1 and Table S2). Due to a higher species richness that was observed in the dataset with forward reads (Fig. S1), we decided to exclusively use this dataset for further analyses. Bioinformatic analysis of the amplicon sequences was performed using the open-source QIIME2 version 2018.4.0 pipeline (https://qiime2.org [37];). Demultiplex raw reads were imported to QIIME2 using ‘qiime tools import’. Primer sequences were removed using the cutadapt plugin [38]. The DADA2 algorithm was used to quality filter and denoise demultiplexed sequences [39]. Subsequently, chimeric sequences were removed using the DADA2 chimera removal. The resulted amplicon sequences variants (ASVs) were taxonomically classified by using the VSEARCH classifier [40] against the reference database Silva v128 [41]. Prior further analysis, all reads assigned to *Cyanobacteria* and mitochondria were removed from the dataset.

The herein used metagenomic dataset (MG-RAST ID: mgm4551030.3) was previously reported [20] in the context of screening for arsenic-related functions. It was obtained from the same lichen population that was used for the present study. The raw data was re-analysed with updated bioinformatic tools to investigate ecological function and ARGs diversity in the lichen holobiont. Shotgun metagenomic reads were subjected to adapter trimming and quality filtering using Trimmomatic and VSEARCH [40, 42]. The filtered reads were used as input files for taxonomic profiling using Kaiju [43] and for assembly using metaSPAdes with default parameters [44]. The filtered reads were mapped back to the assembled contigs using Bowtie2 [45]. The assembled contigs were annotated using the blastx algorithm in DIAMOND [46] against eggNOG version 4.5 database [47] and the manually curated antimicrobial resistance gene database (deepARG) [48] to perform ecological function and antimicrobial resistance genes profiling. To minimize the risk of false positives, reads were defined as ARG-like reads at the cut-off *E* value of 10^−10^ and similarity of 35% as previously described by [49, 50]. FeatureCounts [51] were used to align metagenomic reads to the annotated contigs and to obtain total read numbers, respectively. Amplicon sequences were deposited at the European Nucleotide Archive (ENA) under the project number PRJEB37912.

### Statistical analyses

The R version 1.2 (R Core Team, 2017) was used to perform general statistical analysis and visualize results. Significant differences (*P* < 0.05) of bacterial gene copy numbers were analysed using the non-parametric Kruskall-Wallis test. The ASV tables and taxonomic classifications that were generated with QIIME2 were imported into R via phyloseq [52]. The number of sequences from each amplicon sequencing library was normalized to the lowest number of read counts (1009 reads per sample) by randomly selecting subsets of sequences. A taxonomy summary of the top 100 most abundant ASV at class level was visualized by using the integrated bar plots. Differences in microbial alpha diversity based on the number of identified ASVs and the Shannon index were analysed using the non-parametric Kruskall-Wallis test followed by the paired difference test, Wilcoxon signed-rank test. The beta diversity assessment based on normalized Bray-Curtis dissimilarity matrix was subjected to the Adonis test (999 permutations) to determine the effect of antimicrobial exposure and different dosages on microbial community structures. The distance matrices were visualized using non-metric multidimensional scaling (NMDS) plots. The analyses mentioned above were performed using the R package vegan [53]. We also correlated total and active bacterial community matrix distance through partial Mantel tests (corrected for spatial distance) with 999 permutations. Bacterial genera associated with each antibiotic treatment were identified by LefSe (liner discriminant analysis effect size) as implemented in MicrobiomeAnalyst [54–56]. The threshold for the linear discriminant analysis (LDA) was set to 2 with a *P* value cut-off of 0.05. Finally, the correlation analysis implemented in the ggpubr package [57] was used to calculate Spearman coefficients for correlations between bacterial genera and different antimicrobial compounds.

### Complementary quantification of the *mcr1* gene by qPCR

To quantify AMR activation of the positive responders at mRNA level, we performed a targeted qPCR analysis of the *mcr1* gene. The *mcr1* gene was selected, because it is the only known gene that confers colistin resistance. We therefore expected that it is present in lichen-associated bacteria that thrive under colistin exposure. The qPCR experiments were conducted using the primer pair mcr1FP-mcr1RP as previously described [58]. A *mcr1* standard was obtained from ten-fold serial dilutions of the genomic DNA of a colistin-resistant *Escherichia coli* isolate. The isolate is part of the culture collection of the Department of Internal Medicine, Medical University, of Graz. Extracted cDNA from colistin-treated and control samples was subjected to qPCR analyses using the Rotor-Gene 6000 real-time rotary analyser (Corbett Research) with previously described parameters [58].

## Results

### The *Peltigera* microbiome and antimicrobial resistance genes

**Fig. 1:**
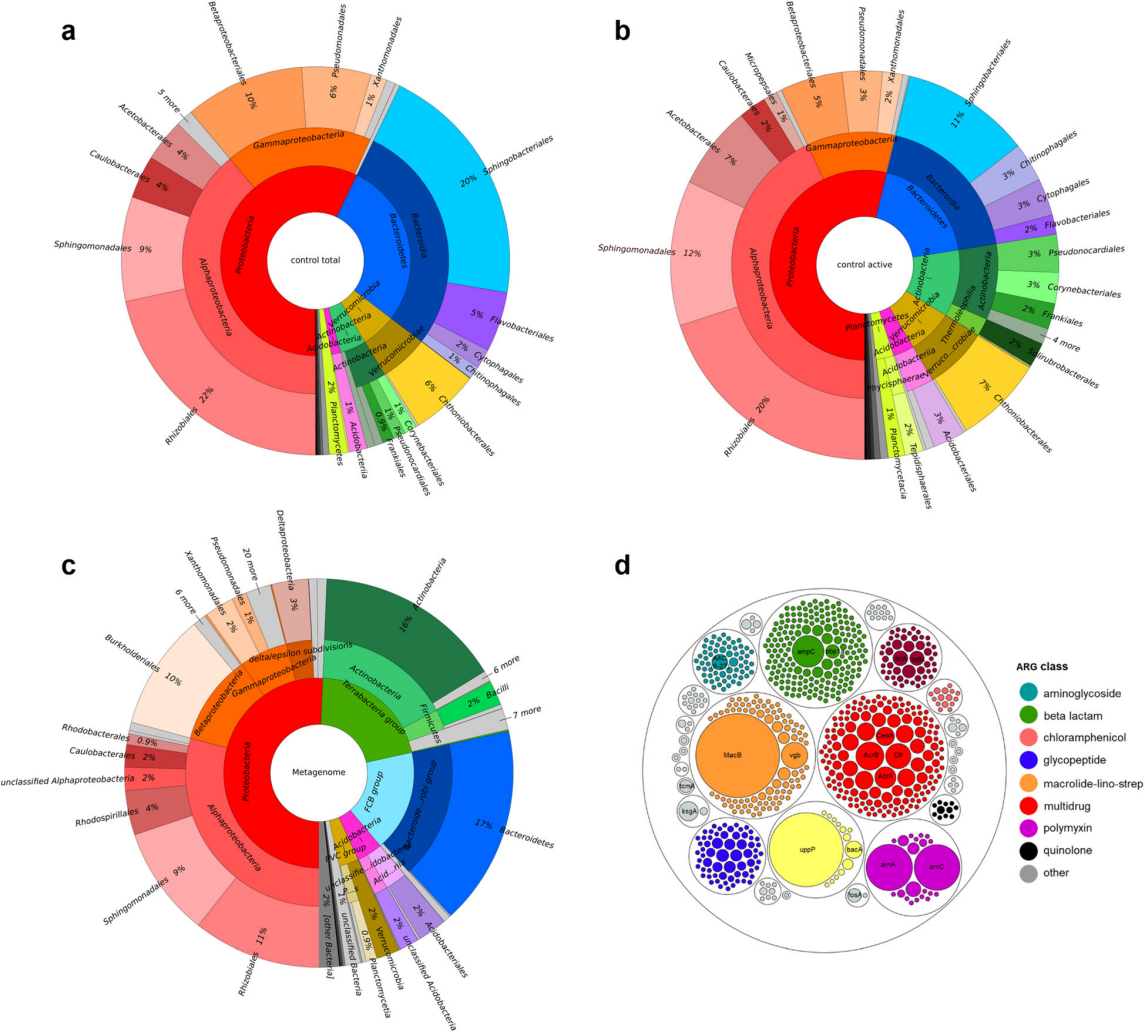
The results of bacterial community (**a**–**c**) and antimicrobial resistance gene profiling (**d**) of the *Peltigera*-associated microbiome are visualized in Krona charts and a circle packing plot. The lichen thallus-associated community was assessed with **a** DNA-based amplicon sequencing, **b** RNA-based amplicon sequencing, and **c** shotgun metagenomic sequencing. An AMR profile was obtained by specific assignments within the deepARG database. Different colours indicate specific ARG classes

A total of 1.67 × 10^6^ (1.63 × 10^4^ per sample mean) high-quality reads were obtained in the amplicon sequencing approach. From the metagenomic dataset, a total of 13.9 × 10^6^ reads were annotated using the eggNOG database, while a total of 3.04 × 10^5^ reads (0.82% of total reads) was assigned to ARGs using the deepARG database. According to the Kaiju classifier, we detected metagenomic contigs that were classified as *Cyanobacteria*. Amplicon libraries were also dominated by *Nostocaceae* (C*yanobacteria* phylum) sequences. These contigs and the respective raw sequences were not evaluated as part of the bacterial community because *Nostoc* represent the well-studied, homogenous phototobiont in *Peltigera*, and they commonly carry only a few distinct ARGs, for example *mtrA* (multidrug resistance gene). After filtering non-target taxa, a total of 8.6 × 10^5^ (8.4 × 10^3^ per sample mean) amplicon sequencing reads were retained and assigned to 3124 bacterial ASVs. Comparison between community assessments on metagenome, DNA amplicon, and RNA amplicon level revealed that the general bacterial community structure showed a certain congruent at class and order level across the dataset regardless of different approaches (Fig. 1a–c). *Alphaproteobacteria* were the most dominant class in the *Peltigera-*associated microbiome in the non-treated samples with a relative abundance (RA) of 29–43%. The other predominant classes were *Bacteroidetes* (17–28%), and *Gammaproteobacteria*/*Betaproteobacteria* (9–17%). Taxonomic analysis revealed four highly abundant orders, i.e., *Rhizobiales* (17.6%, average RA from metagenomic and amplicon sequencing dataset), *Sphingobacteriales* (12.3%), *Sphingomodales* (10.3%), and *Betaproteobacteriales* (8.7%).

We conducted a general functional analysis, which focused on functions that could directly affect the symbiosis. In the overall dataset, the majority of metagenomic reads (65%) was assigned to bacterial proteins. Therein, we detected numerous reads (1.4%) assigned to Ton and Tol transport systems which are involved in iron uptake. Many of these reads were derived from *Sphingomonas*, *Methylobacterium*, and *Mucilaginibacter*. Genes that are involved in vitamin production such as cobalamin biosynthesis protein and folate metabolism (0.3%) were also detected within contigs derived from these taxa. In addition, bacterial porin proteins such as carbohydrate-selective porin and aquaporin that may be involved in carbohydrate metabolism and drought stress were also detected (0.03%). Using the eggNOG database, we found that a high number of bacterial reads (2.6%) were assigned to defence mechanism function. The majority of those proteins (25.5%) were annotated as part of an ABC-transport system.

More specific profiling of antimicrobial resistance genes in the metagenomic dataset against the deepARG database resulted in the detection of 728 ARGs spanning 30 antimicrobial classes (Fig. 1d). Most of the identified ARGs originated from *Proteobacteria* (28% of *Alphaproteobacteria* and 20% of *Beta*/*Gammaproteobacteria*, Fig. S2). A total of 80.5% detected ARGs were classified to macrolide-lincosamide-stretogramin (MLS) multidrug classes, bacitracin, beta lactam, and polymyxin. This finding indicated a high diversity, but a low abundance, of ARGs embedded in the lichen metagenome (Fig. 1d).

### Responses to exposure to antimicrobials at phenotype and genotype level (richness and diversity)

Antimicrobial treatments resulted in phenotypic changes to the exposed *Peltigera* thalli. A change of colour from drab grey-green to dark brown in lichen samples treated with the full dosage of the alkylpyrazine was observed after 3 days of continuous exposure in comparison to the control (Fig. S3). A similar phenotypic change was also observed in samples treated with the full dosage of glyphosate after 5 days, which became more obvious after 8 days of continuous exposure. We did not observe a change of colour in lichen thalli that were treated with sub-therapeutic dosages of alkylpyrazine and glyphosate as well as colistin and tetracycline treated samples in comparison to the control during the whole experiment. These changes indicate that the naturally occurring cyanobacteria were negatively affected by the alkylpyrazine and glyphosate treatments due to algicidal properties of these antimicrobials.

Different exposures to antimicrobials and their respective dosages affected bacterial richness (*P* < 0.001, *P* = 0.010, respectively, Table S3) according to the Shannon diversity index (*H*), whereas the assessment type (DNA- or RNA-based amplicons) did not have any effect on the alpha diversity (*P* = 0.632, Table S3). When analysed separately, each of the employed dosages of alkylpyrazine and glyphosate showed no significant changes in bacterial richness when compared to the untreated control group (*P* > 0.05, Table S3). In contrast, highly significant changes were observed for the colistin and tetracycline treatments (*P* < 0.001, Table S3). Increased dosage of these antimicrobial substances resulted in substantially reduced bacterial richness. The highest impact was observed in the samples exposed to the full dosages of colistin (*H*’ = 1.7 and *H*’ = 1.7—total and active bacteria, Table S4) and tetracycline (*H*’ = 1.4 and *H*’ = 2.5) in comparison to non-treated samples (*H*’ = 4.3 and *H*’ = 4.3).

**Fig. 2:**
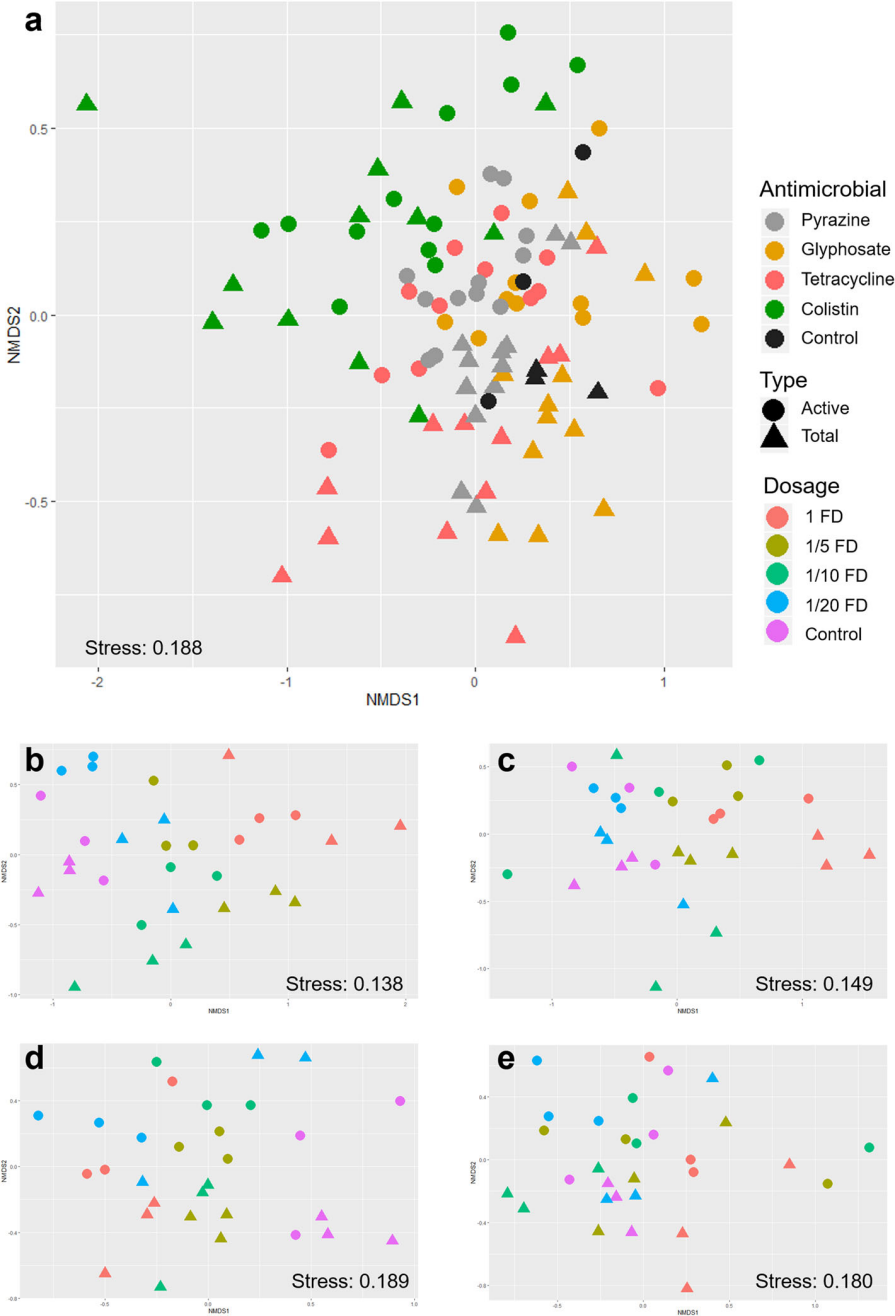
Community clustering of bacterial composition (total and active bacterial community) in lichens treated with different antimicrobial compounds is visualized in two-dimensional Bray-Curtis NMDS plots. The plots are based on **a** all datasets, **b** colistin dataset, **c** tetracycline dataset, **d** glyphosate dataset, and **e** alkylpyrazine dataset

To investigate the impact of the antimicrobial exposure on the bacterial community structure, beta diversity analysis was performed using Bray-Curtis matrix distance in combination with Adonis and visualized using a non-metric multidimensional scaling (NMDS) plot. The antimicrobial type was found to be the main driver of the bacterial community structure (*R*^2^ = 0.265, *P* = 0.001, Table 2, Fig. 2a), whereas the other factors, such as antimicrobial dosage or type of community (total or active bacterial fraction) only explained a small amount of the variation (*P* = 0.001; *R*^2^ = 0.076 and *R*^2^ = 0.043, respectively). A complementary Mantel test showed a highly significant correlation of both, the total bacterial community and the active community (*P* = 0.001, *R* = 0.719). This indicated a high congruent between these approaches. When the data was separated according to the antimicrobial substance, we observed that antimicrobial dosage in each dataset had a substantial impact on bacterial community structure (*P* = 0.001, *R*^2^ = 0.255–0.571). In ordination space, a clear clustering was observed between treated and non-treated samples where samples that were treated with higher dosage are further apart from non-treated samples (Fig. 2b–e). A noteworthy result was that a higher impact of antimicrobial dosage was observed in the colistin dataset (*R*^2^ = 0.571) compared to other datasets.

**Table 2:** **Effect of antimicrobial treatment, dosages, and sample type (DNA or RNA) on bacterial community structure (**β-diversity). A complementary statistical analysis was conducted in order to identify factors with a significant effect on the bacterial community

Factor	Microbial community similarities	
	*R*^2^ value	*P* value
**All datasets**		
Antimicrobials	0.265	0.001*
Dosage	0.076	0.001*
Type	0.043	0.001*
**Colistin dataset**		
Dosage (D)	0.528	0.001*
Type (T)	0.069	0.001*
D × T	0.131	0.001*
**Tetracycline dataset**		
Dosage (D)	0.471	0.001*
Type (T)	0.065	0.007*
D × T	0.097	0.087
**Alkylpyrazine dataset**		
Dosage (D)	0.340	0.001*
Type (T)	0.134	0.001*
D × T	0.128	0.011*
**Glyphosate dataset**		
Dosage (D)	0.255	0.001*
Type (T)	0.070	0.001*
D × T	0.099	0.769

*Significant differences (*P* ≤ 0.05) were assessed with the Adonis test

### Antimicrobial exposure induces changes in bacterial community composition without altering bacterial abundances

To investigate the total and active bacterial abundance of the lichen-associated bacteria after antimicrobial exposure, a quantitative polymerase chain reaction (qPCR) approach with specific bacterial primers, targeting the 16S ribosomal RNA gene was performed. Total bacterial rRNA gene abundance ranged between 1.02 × 10^7^ and 1.90 × 10^8^ 16S rRNA gene copies per ng extracted DNA whereas active bacterial rRNA gene abundance ranged between 4.40 × 10^3^ and 6.35 × 10^4^ 16S rRNA gene copies per ng extracted RNA (Table S5). Overall, statistical significance tested using the Kruskal-Wallis test showed no effect of antimicrobial treatment on bacterial abundance (*P* > 0.05).

**Fig. 3:**
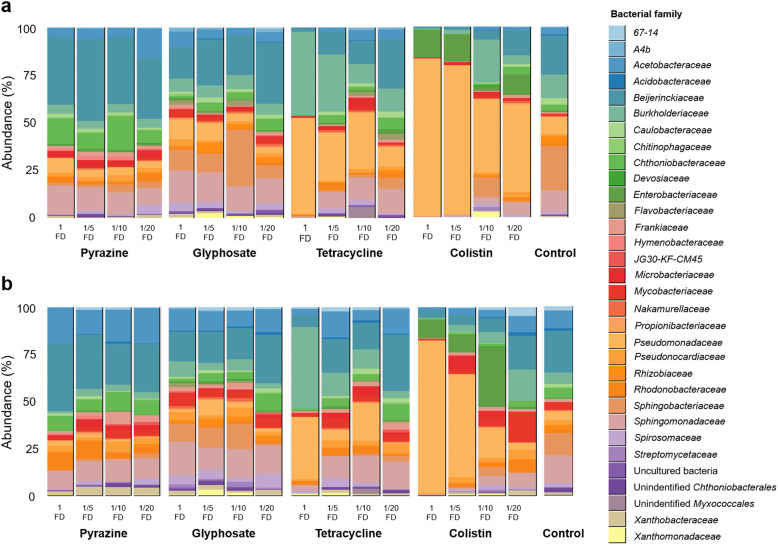
Relative abundance plot of the top 100 most abundant bacterial families in lichen samples with or without antimicrobial treatment. A differential assessment of the **a** total bacterial community and **b** active bacterial community was conducted. For each included treatment, antimicrobials at their full dosage as well as three sub-therapeutic dosages were applied

In order to visualize taxonomic composition of the lichen holobiont after antimicrobial exposures, bar plots showing the 100 most abundant bacterial ASVs were constructed (Fig. 3). Distinct taxonomical changes were observed after antimicrobial exposure depending on the type of antimicrobial and their dosages. Taxonomical shifts after exposure to colistin were more similar to tetracycline exposure whereas glyphosate exposure showed a more similar taxonomical shift to alkylpyrazine exposure.

Most remarkably, the abundances of *Pseudomonadaceae* family (*Gammaproteobacteria*), increased in response to colistin exposure. The relative abundance of *Pseudomonadaceae* gradually increased in response to colistin exposure which reached up to 79.6% and 80.3% in the total and active bacterial fraction, respectively, when exposed to the full dosage of colistin. In contrast, this taxon represented only 5.9 and 3.3% (total and active bacterial fraction, respectively) in non-treated samples. A similar pattern was observed after exposure to tetracycline where the relative abundance increased up to 49.4% and 31.1% in the treatment with the full dosage. In contrast, the relative abundances of *Sphingobacteriaceae* as well as *Sphingomonadaceae* decreased to below 0.2% and 0.6% after full dosage exposure of these antimicrobial substances. Taxonomical shifts in the bacterial families were also observed after alkylpyrazine exposure with an increase in *Beijerinckiaceae*, ranging from 26.5% and 20.8% (total and active bacterial fraction, respectively), with the lowest concentration of alkylpyrazine, to 30.4% and 31.1% in the full dosage, in comparison to non-treated samples (17.3% and 16.4%). Overall, exposure to antimicrobial substances at different dosages induced shifts in the bacterial community structure (Fig. 3a, b). Moreover, an indication of microbial imbalance due to selectively enriched low abundant taxa was observed in samples treated with colistin and tetracycline.

### Putative roles of the identified bacterial responders

**Fig. 4:**
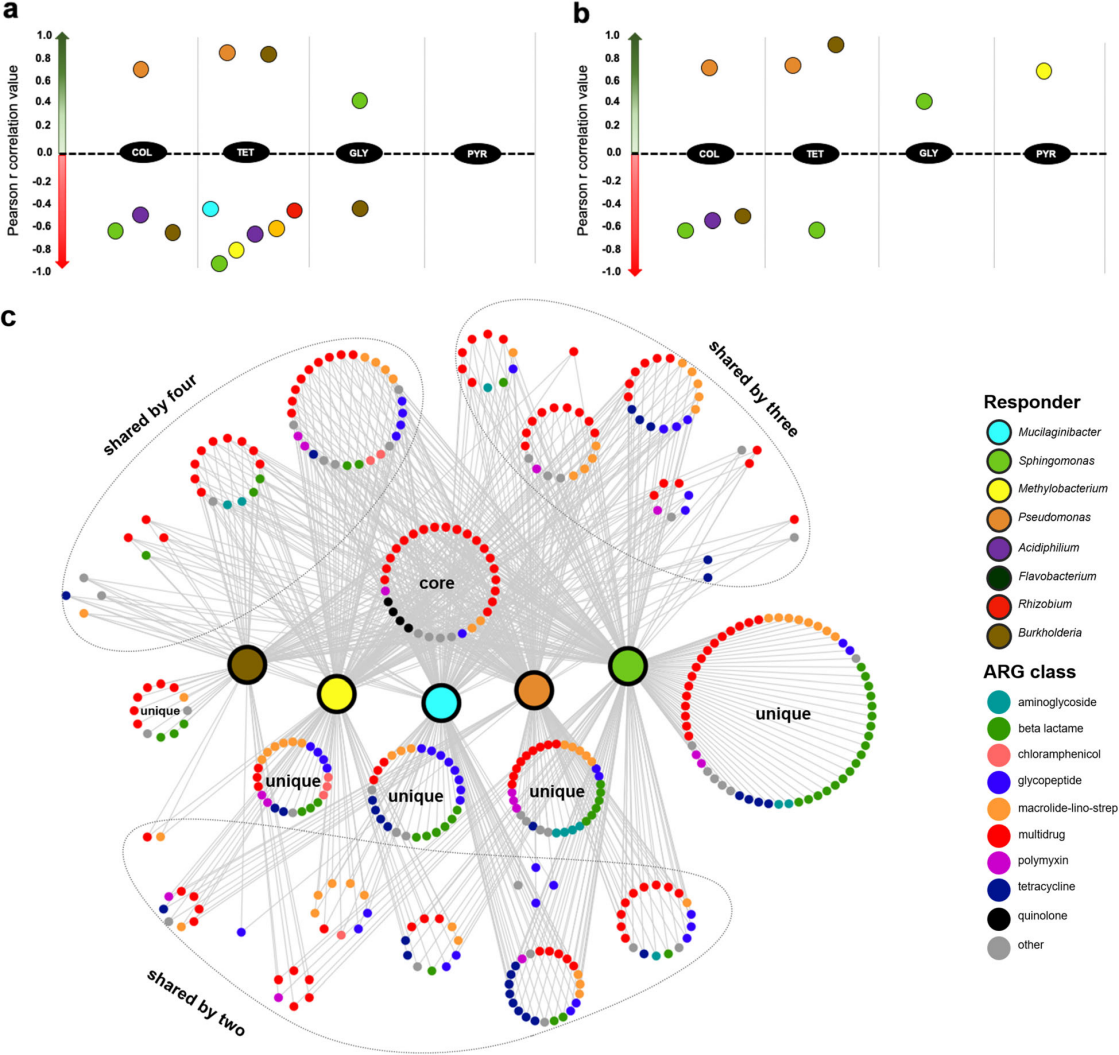
Correlation analysis between relative abundances of bacterial genera and antimicrobial dosages (**a**, **b**) and network visualization of shared and unique antimicrobial resistance genes (ARGs) from selected bacterial responders (**c**). Responders (indicated with different colours) are shown in the **a** total bacterial community and **b** active bacterial community. Only correlations with *P* < 0.05 are displayed. The nodes in the network are coloured according to ARG classes. COL, colistin; TET, tetracycline; GLY, glyphosate; PYR, 5-isobutyl-2,3-dimethylpyrazine

Calculation of linear discriminant analysis effect size (LEfSe) was performed to identify taxa that were significantly affected by antimicrobial treatments. The analysis indicated that 15 and 12 bacterial genera were affected in the total and active bacterial community of antimicrobial-treated and non-treated samples, respectively (Fig. S4). *Pseudomonas* and *Curtobacterium* were consistently enriched under colistin exposure and *Burkholderia* was consistently enriched under tetracycline exposure (Fig. S4). The analysis also indicated that *Methylobacterium*, *Acidiphilium*, and an unidentified member of *Beijerinckiaceae* were consistently enriched under alkylpyrazine exposure. In complementary analyses, we statistically examined correlations between the relative abundance of bacterial genera and antimicrobial concentrations. To minimize spurious correlation, we selected only bacterial genera with a relative abundance above 2% in the whole dataset. *Acidiphilium*, *Burkholderia,* and *Sphingomonas* were found to consistently negatively correlate to increase dosage of colistin in both, total and active bacterial community. The latter taxon was also negatively correlated with increasing tetracycline dosage. Several genera, i.e., *Acidiphilium, Flavobacterium*, *Mucilaginibacter*, *Methylobacterium*, and *Rhizobium* were identified to also negatively correlate to increased dosages of tetracycline in the total bacterial community dataset. We further identified bacterial genera that were positively correlated to increased antimicrobial dosage in both, the total and the active bacterial community (*P* < 0.05, Fig. 4a, b). The relative abundance of *Pseudomonas* was found to correlate to increased dosage of colistin and tetracycline in the total and active bacterial community. *Burkholderia* was found to correlate to an increased dosage of tetracycline while *Sphingomonas* was correlated to an increased dosage of glyphosate (Fig. 4a, b).

To address the question why distinct responders could thrive under pressure caused by specific antimicrobials, we investigated contigs that were assigned to each of the responders from the metagenome dataset and compared the presence/absence of specific antimicrobial resistance genes. From the detected ARGs in the *Peltigera* metagenome, a network of co-occurring ARGs was constructed to visualize shared and unique ARGs of the selected responders (Fig. 4c). A high proportion of multidrug resistance and quinolone were shared between the responders. *Sphingomonas* (*n* = 102), *Pseudomonas* (*n* = 83), and *Methylobacterium* (*n* = 69) had a higher number of multidrug resistance genes in comparison to other responders such as *Burkholderia* (*n* = 47) and *Rhizobium* (*n* = 51). Moreover, a higher number of multidrug resistance genes in *Sphingomonas* and *Methylobacterium* contigs may explain how responders could thrive during exposure to non-clinical antimicrobial substances, glyphosate and alkylpyrazine, respectively. Despite the high occurrence of shared ARG genes, *mcr1*, a colistin resistance gene, was detected in *Pseudomonas*-derived contigs indicating that this gene may be responsible for its thriving under colistin exposure. When copy numbers of the *mcr1* transcript were analysed via qPCR in colistin-treated and non-treated samples, significantly (*P* = 0.007) higher transcript numbers were found in colistin-treated samples irrespective of the dosage in comparison to non-treated samples (Table S6).

All positive and negative responders were shown to carry tetracycline resistance genes. A higher number of tetracycline resistance genes was found in negative responders *Mucilaginibacter* (*n* = 19) and *Sphingomonas* (*n* = 22) derived contigs in comparison to the positive responders, i.e., *Burkholderia* (*n* = 3) and *Pseudomonas* (*n* = 4). We also detected *tetAB(46)* and *tetAB(60)* that was only shared between *Mucilaginibacter* and *Sphingomonas.* These genes encode ABC transporters that confer resistance to tetracycline.

## Discussion

Our data highlights that the natural microbiome of *Peltigera* comprises highly diverse and low abundant intrinsic ARGs, which provide a retrievable basis to cope with antimicrobial pressure. Similar to other relatively pristine environments, ARGs were found to be ubiquitous and to harbour a high number of different efflux pump systems [13, 59, 60]. In nature, ARGs fulfil various roles and are commonly involved in processes such as detoxification and molecular signalling. However, the same mechanisms can also serve as an essential feature of nosocomial pathogens to overcome high (toxic) antimicrobial concentrations that are found in clinical settings [59, 61]. The high diversity of ARGs that was detected in the present study, reflects the natural complexity of microbial communities that are commonly associated with lichens, and have previously been shown to provide metabolic versatility that facilitates plasticity of the lichen holobiont [13, 62].

Our study provides the first detailed insights into community-level response to antimicrobial exposure in a pristine system. In agreement to previous reports and, as expected, a higher impact of antimicrobial exposure was observed on the bacterial community structure compared to the bacterial abundance [26, 30]. We observed a specific shift in the taxonomic composition and community structure of native bacteria as a response to antimicrobial exposure. Despite minor variations, comparable and congruent alpha and beta diversity results between DNA- and RNA-based amplicon sequencing indicated that both of the approaches reflected how antimicrobial exposure shaped the bacterial community. Both approaches led to the identification of mostly overlapping responder taxa. Moreover, the similarity between the DNA and RNA approach indicates that the majority of bacteria in lichen was active. We observed a relatively lower effect after exposure to non-clinical antimicrobial compounds, i.e*.*, glyphosate and alkylpyrazine despite their broad activity spectrum compared to the clinical antimicrobial compounds, i.e., colistin and tetracycline. This indicated that the bacterial community is more resilient towards non-clinical antimicrobials. We hypothesize that the diversity of unspecific multidrug efflux pumps that are shared among *Peltigera*-associated bacteria may play a major role in the observed resilience. Distinct taxa such as *Sphingomonas* and *Methylobacterium* are equipped with a high number of multidrug resistance features, which are likely important for their resilience towards non-clinical antimicrobial compounds. Multidrug efflux pumps, especially ABC transporters, are known to contribute to herbicide resistance [63, 64]. Despite this, there are no studies reporting resistance genes against pyrazines, producers of these compounds can be frequently found in nature and more specifically in the microbiota of other lichens [65, 66]. Thus, *Peltigera*-associated bacteria may likely encounter these antimicrobial compounds in nature whereby multidrug efflux pumps likely provide the best means for detoxification [61].

Certain taxa in the lichen microbiome that naturally occur in low abundances but are equipped with specific resistance features, increased in response to colistin and tetracycline. Antimicrobial exposure might have created a temporary ‘biological vacuum’ as a result of the reduction of bacterial diversity and therefore created a new niche for more resilient bacteria (responders) for recolonization and bloom [67, 68]. *Pseudomonas* consistently increased in response to colistin and tetracycline. The *mcr1* gene, which encodes a phosphoethanolamine transferase, was found among *Pseudomonas*-assigned contigs in the metagenomic dataset and provides an explanation for the resilience against colistin. The *mcr1* transcripts were also higher in colistin-treated samples in comparison to non-treated samples when assessed with a complementary qPCR approach. This gene constitutes the only known mechanism to confer colistin resistance by altering antimicrobial-specific binding sites (reviewed in [69–71]). It remains unclear how the positive responders, i.e., *Pseudomonas* and *Burkholderia* could thrive under tetracycline exposure, because negative responders were shown to also harbour tetracycline resistance genes. Nevertheless, it is worth to mention that the presence of genes in a metagenomic library do not necessarily imply their functional expression [72]. This is important in the context of our study since we detected a high number of tetracycline resistance genes in *Sphingomonas* and *Mucilaginibacter* despite the observed negative effect of tetracycline exposure on their relative abundance. Nevertheless, other factors such as increased bacterial resilience through biofilm formation, the host response and nutrient availability that were not assessed in this study, might be involved in increased abundance of distinct taxa under specific antimicrobial exposure [73, 74]. Therefore, further studies based on metatranscriptomic and metaproteomic approaches will be needed to identify genes, proteins, and pathways that are associated with bacterial community responses during antimicrobial exposure.

We showed that naturally dominant taxa, such as *Sphingomonas* and *Mucilaginibacter*, were negatively correlated to increased concentrations of clinical antimicrobials. Lichens are known to harbour bacteria with functional guilds that play an essential role as probiotics and detoxifiers [18]. Dominant taxa, such as *Sphingomonas* and *Mucilaginibacter* that encoded for various transport machineries, such as Ton- and Tol-dependent transport as well as porins, are suggested to play important roles in iron metabolism and transport as part of a survival strategy in the lichen holobiont [14, 75]. Following antimicrobial exposure, it was observed that the native microbiota may be restored to the initial composition; however, the restoration remains often incomplete (reviewed in [76]). Therefore, collateral damage of dominant and crucial taxa from prolonged exposure to antimicrobials may disrupt the fine-tuned symbiotic interplay in lichens even if they harbour resistant taxa. In the present study, we also observed a bloom of low abundant taxa that carry features that are known to confer colistin resistance. This is relevant in the context of potential spread of natural AMRs to clinical settings, which currently rely on this antibiotic. Lichens that cover up to 8% of the total terrestrial surface [77] may be increasingly affected by anthropogenic activities in the future such as overuse of antimicrobial substances [78]; the use of antimicrobials for agricultural purposes is predicted to increase at least 99% by 2030 [79]. Increased antimicrobial pressure in natural AMR reservoirs may increase the risk for resistance transmission to opportunistic human pathogens [59, 80].

## Conclusion

Lichens are ideal model organisms to mechanistically study how antimicrobial exposure affects native microbiota due to their well-defined, highly diverse bacterial colonizers. All antimicrobial substances showed an impact on the microbiome and we identified distinct positive as well as negative responders. Microbial dysbiosis caused by exogenic antimicrobials can result in a bloom of naturally low abundant taxa (positive responders) with specific AMR features. Bacteria assigned to the genera *Pseudomonas, Sphingomonas, Burkholderia,* and *Methylobacterium* were identified as positive responders; many species of these genera are already well-known nosocomial pathogens in clinical environments. The findings of the present study indicate that in situ exposure of microbial communities can facilitate the identification of AMR-carriers with resistance features of opportunistic human pathogens and is thus a valuable tool to explore their emergence. Moreover, these and future findings may be translatable into new management strategies for AMR-affected environments, e.g., alternating use of different antimicrobials in clinical settings to reduce specific antimicrobial pressure that was shown to result in a bloom of distinct resistance carriers.

## Supplementary Information

**Additional file 1.** Table S1. Comparison of the alpha diversity in forward and paired-end read datasets. Table S2. Comparison of the beta diversity in forward and paired-end read datasets. Table S3. Effect of antimicrobial treatment, dosages, and sample type (DNA or RNA) on lichen associated bacterial richness (alpha diversity) according to the Shannon diversity index. Table S4. Lichen-associated bacterial richness (alpha diversity) according to the Shannon diversity index following exposure to antimicrobial compounds. Table S5. Real time qPCR-based assessment of total bacterial 16S rRNA gene copy numbers in lichens after exposure to different antimicrobial substances. Table S6. Real time qPCR-based assessment of *mcr*-1 resistance gene copy numbers in lichens after exposure to colistin. Figure S1. Rarefaction curves showing the number of ASVs that were observed in lichens treated with different antimicrobial compounds. Rarefaction curves are based on (a-e) the forward-read-only dataset and (f-j) the paired-end read dataset and derived from the (a,f) colistin (b,g), tetracycline (c,h), alkylpyrazine (d,i) and glyphosate (e,j) treatments. Figure S2. Identification of carriers of the detected antimicrobial resistance genes. The annotation was conducted using the metagenome classifier Kaiju and visualized with the integrated bubble plot tool. Figure S3. Phenotypes of lichen samples that were treated with full dosages of antimicrobial compounds i.e. colistin, tetracycline, glyphosate, and alkylpyrazine in comparison to the untreated control. Representative lichen samples were documented on day 3, 5 and 8 after the first spray application of the antimicrobial substances. Figure S4. Identification of responders to different antimicrobial compounds by LEfSe (Linear discriminant analysis effect size). The analyses are based on (a) the total and (b) the active bacterial community. Only bacterial genera with a LDA score above 2 and cut-off *P* values below 0.05 were included.

## Data Availability

Raw sequencing data for each sample used in this study was deposited at the European Nucleotide Archive (ENA) in the FASTQ format and is available under the Bioproject accession number PRJEB37912.

## Abbreviations

AMR: Antimicrobial resistance
ARG: Antimicrobial resistance gene
FD: Full dosage
DNA: Deoxyribonucleic acid
RNA: Ribonucleic acid
cDNA: Complementary DNA
PCR: Polymerase chain reaction
qPCR: Real-time (quantitative) polymerase chain reaction
ASV: Amplicon sequences variant
NMDS: Non-metric multidimensional scaling
MLS: Macrolide-lincosamide-stretogramin
