# Supplementary material for: Antimicrobial-specific response from resistance gene carriers studied in a natural, highly diverse microbiome
Source: Microbiome. 2021 Jan 27;9:29. doi: 10.1186/s40168-020-00982-y (PMC7841911; doi:10.1186/s40168-020-00982-y)
Supplement: Supplementary file 2 — Additional file 1. Table S1. Comparison of the alpha diversity in forward and paired-end read datasets. Table S2. Comparison of the beta diversity in forward and paired-end read datasets. Table S3. Effect of antimicrobial treatment, dosages, and sample type (DNA or RNA) on lichen associated bacterial richness (alpha diversity) according to the Shannon diversity index. Table S4. Lichen-associated bacterial richness (alpha diversity) according to the Shannon diversity index following exposure to antimicrobial compounds. Table S5. Real time qPCR-based assessment of total bacterial 16S rRNA gene copy numbers in lichens after exposure to different antimicrobial substances. Table S6. Real time qPCR-based assessment of mcr-1 resistance gene copy numbers in lichens after exposure to colistin. Figure S1. Rarefaction curves showing the number of ASVs that were observed in lichens treated with different antimicrobial compounds. Rarefaction curves are based on (a-e) the forward-read-only dataset and (f-j) the paired-end read dataset and derived from the (a,f) colistin (b,g), tetracycline (c,h), alkylpyrazine (d,i) and glyphosate (e,j) treatments. Figure S2. Identification of carriers of the detected antimicrobial resistance genes. The annotation was conducted using the metagenome classifier Kaiju and visualized with the integrated bubble plot tool. Figure S3. Phenotypes of lichen samples that were treated with full dosages of antimicrobial compounds i.e. colistin, tetracycline, glyphosate, and alkylpyrazine in comparison to the untreated control. Representative lichen samples were documented on day 3, 5 and 8 after the first spray application of the antimicrobial substances. Figure S4. Identification of responders to different antimicrobial compounds by LEfSe (Linear discriminant analysis effect size). The analyses are based on (a) the total and (b) the active bacterial community. Only bacterial genera with a LDA score above 2 and cut-off P values below 0.05 were included. [file 40168_2020_982_MOESM2_ESM.docx]

**Supplementary Material**

**Antimicrobial-specific response from resistance gene carriers studied in a natural, highly diverse microbiome**

**Wisnu Adi Wicaksono^1^, Peter Kusstatscher^1^, Sabine Erschen^1^**, **Tamara Reisenhofer-Graber ^1^, Martin Grube^2^, Tomislav Cernava^1^, Gabriele Berg^1^**

Author affiliations

*^1^Institute of Environmental Biotechnology, Graz University of Technology, Graz, Austria*

*^2^Institute of Biology, University of Graz, Graz, Austria*

Corresponding author:

Tomislav Cernava, Graz University of Technology, Graz

Email: tomislav.cernava@tugraz.at

**Authors’ email addresses:** wisnu.wicaksono@tugraz.at, peter.kusstatscher@tugraz.at, sabine.erschen@tugraz.at, reisenhofer-graber@student.tugraz.at, martin.grube@uni-graz.at, tomislav.cernava@tugraz.at, gabriele.berg@tugraz.at

**Short title:** The lichen resistome

**Submitted to:** Microbiome

**Keywords**

Lichen microbiota, *Peltigera* *polydactylon*, Antimicrobial resistance, Metagenomic mining, Genome recovery

**Table S1. Comparison of the alpha diversity in forward and paired-end read datasets.**

| **Factor** | **Alpha diversity** | | | |
| --- | --- | --- | --- | --- |
|  | **Forward read**  **dataset** |  | **Paired-end read dataset** |  |
|  | ***P* value** |  | ***P* value** |  |
| **All datasets** |  |  |  |  |
| Antimicrobials | <0.001* |  | <0.001* |  |
| Dosage | 0.010* |  | 0.010* |  |
| Type | 0.632 |  | 0.886 |  |
|  |  |  |  |  |
| **Colistin dataset** |  |  |  |  |
| Dosage | <0.001* |  | <0.001* |  |
| Type | 0.576 |  | 0.852 |  |
|  |  |  |  |  |
| **Tetracycline dataset** |  |  |  |  |
| Dosage | <0.001* |  | <0.001* |  |
| Type | 0.494 |  | 0.633 |  |
|  |  |  |  |  |
| **Alkylpyrazine** **dataset** |  |  |  |  |
| Dosage | 0.073 |  | 0.076 |  |
| Type | 0.206 |  | 0.071 |  |
|  |  |  |  |  |
| **Glyphosate dataset** |  |  |  |  |
| Dosage | 0.227 |  | 0.298 |  |
| Type | 0.633 |  | 0.633 |  |

*Significant differences (*P* ≤ 0.05) were assessed with the Kruskal-Wallis test.

**Table S2. Comparison of the beta diversity in forward and paired-end read datasets.**

| **Factor** | **Microbial community similarities** | | | | |
| --- | --- | --- | --- | --- | --- |
|  | **Forward read**  **dataset** | |  | **Paired end read dataset** | |
|  | *R^2^* value | *P* value |  | *R^2^* value | *P* value |
| **All datasets** |  |  |  |  |  |
| Antimicrobials | 0.265 | 0.001* |  | 0.227 | 0.001* |
| Dosage | 0.076 | 0.001* |  | 0.067 | 0.001* |
| Type | 0.043 | 0.001* |  | 0.039 | 0.001* |
|  |  |  |  |  |  |
| **Colistin dataset** |  |  |  |  |  |
| Dosage (D) | 0.528 | 0.001* |  | 0.476 | 0.001* |
| Type (T) | 0.069 | 0.001* |  | 0.060 | 0.001* |
| D * T | 0.131 | 0.001* |  | 0.143 | 0.001* |
|  |  |  |  |  |  |
| **Tetracycline dataset** |  |  |  |  |  |
| Dosage (D) | 0.471 | 0.001* |  | 0.415 | 0.001* |
| Type (T) | 0.065 | 0.007* |  | 0.061 | 0.004* |
| D * T | 0.097 | 0.087 |  | 0.109 | 0.112 |
|  |  |  |  |  |  |
| **Alkylpyrazine** **dataset** |  |  |  |  |  |
| Dosage (D) | 0.340 | 0.001* |  | 0.293 | 0.001* |
| Type (T) | 0.134 | 0.001* |  | 0.119 | 0.001* |
| D * T | 0.128 | 0.011* |  | 0.133 | 0.011* |
|  |  |  |  |  |  |
| **Glyphosate dataset** |  |  |  |  |  |
| Dosage (D) | 0.255 | 0.001* |  | 0.239 | 0.001* |
| Type (T) | 0.07 | 0.001* |  | 0.067 | 0.003* |
| D * T | 0.099 | 0.769 |  | 0.115 | 0.474 |

*Significant differences (*P* ≤ 0.05) were assessed with the Adonis test.

**Table S3. Effect of antimicrobial treatment, dosages, and sample type (DNA or RNA) on lichen associated bacterial richness (alpha diversity) according to the Shannon diversity index.**

| **Factor** | ***P* value^*^** |
| --- | --- |
| **All datasets** |  |
| Antimicrobials | <0.001 |
| Dosage | 0.010 |
| Type | 0.632 |
|  |  |
| **Colistin dataset** |  |
| Dosage | <0.001 |
| Type | 0.576 |
|  |  |
| **Tetracycline dataset** |  |
| Dosage | <0.001 |
| Type | 0.494 |
|  |  |
| **Alkylpyrazine** **dataset** |  |
| Dosage | 0.073 |
| Type | 0.206 |
|  |  |
| **Glyphosate dataset** |  |
| Dosage | 0.227 |
| Type | 0.633 |

^*^ Significance assessments were conducted with the Kruskal-Wallis test.

**Table S4. Lichen-associated bacterial richness (alpha diversity) according to the Shannon diversity index following exposure to antimicrobial compounds.**

| Antimicrobial | Dosage | Shannon diversity index* | |  |
| --- | --- | --- | --- | --- |
|  |  | Total bacteria | Active bacteria | |
| Control | None | 4.3 | 4.3 | |
|  |  |  |  | |
| Colistin | Full dosage | 1.7 | 1.7 | |
|  | 1/5 full dosage | 2.1 | 3.0 | |
|  | 1/10 full dosage | 3.6 | 2.9 | |
|  | 1/20 full dosage | 3.4 | 4.3 | |
|  |  |  |  | |
| Tetracyline | Full dosage | 1.4 | 2.5 | |
|  | 1/5 full dosage | 3.6 | 3.8 | |
|  | 1/10 full dosage | 4.0 | 4.1 | |
|  | 1/20 full dosage | 4.3 | 4.6 | |
|  |  |  |  | |
| Alkylpyrazine | Full dosage | 4.0 | 3.7 | |
|  | 1/5 full dosage | 4.3 | 4.1 | |
|  | 1/10 full dosage | 4.1 | 4.3 | |
|  | 1/20 full dosage | 4.4 | 3.8 | |
|  |  |  |  | |
| Glyphosate | Full dosage | 4.7 | 4.7 | |
|  | 1/5 full dosage | 4.4 | 4.5 | |
|  | 1/10 full dosage | 4.1 | 4.8 | |
|  | 1/20 full dosage | 4.5 | 4.1 | |

* Values are means of 3 biological replicates for each treatment.

**Table S5.** Real time qPCR-based assessment of total bacterial 16S rRNA gene copy numbers in lichens after exposure to different antimicrobial substances.

| Antimicrobial | Dosage | Gene copy number per ng nucleic acid | |
| --- | --- | --- | --- |
|  |  | Total | Active |
| Control | None | 6.8 x 10^7^ | 5.6 x 10^4^ |
|  |  |  |  |
| Colistin | 1/20 FD | 1.9 x 10^8^ | 1.1 x 10^5^ |
|  | 1/10 FD | 3.6 x 10^7^ | 8.4 x 10^3^ |
|  | 1/5 FD | 4.7 x 10^7^ | 4.4 x 10^3^ |
|  | FD | 1.0 x 10^7^ | 6.4 x 10^5^ |
|  |  |  |  |
| Tetracycline | 1/20 FD | 1.8 x 10^8^ | 1.7 x 10^4^ |
|  | 1/10 FD | 1.9 x 10^8^ | 1.2 x 10^4^ |
|  | 1/5 FD | 1.7 x 10^8^ | 1.4 x 10^4^ |
|  | FD | 1.6 x 10^8^ | 1.9 x 10^5^ |
|  |  |  |  |
| Glyphosate | 1/20 FD | 3.3 x 10^7^ | 7.3 x 10^4^ |
|  | 1/10 FD | 3.2 x 10^7^ | 3.0 x 10^5^ |
|  | 1/5 FD | 2.8 x 10^7^ | 4.1 x 10^4^ |
|  | FD | 8.3 x 10^7^ | 6.3 x 10^4^ |
|  |  |  |  |
| Alkylpyrazine | 1/20 FD | 3.1 x 10^7^ | 6.4 x 10^4^ |
|  | 1/10 FD | 8.4 x 10^7^ | 5.1 x 10^4^ |
|  | 1/5 FD | 3.5 x 10^7^ | 6.0 x 10^4^ |
|  | FD | 7.4 x 10^7^ | 8.2 x 10^3^ |

**Table S6.** Real time qPCR-based assessment of mcr-1 resistance gene copy numbers in lichens after exposure to colistin

| Antimicrobial | Dosage | Gene copy number per ng nucleic acid^*^ |
| --- | --- | --- |
| Control | None | 1.2 x 10^3^ a |
|  |  |  |
| Colistin | 1/20 FD | 4.4 x 10^4^ b |
|  | 1/10 FD | 1.4 x 10^4^ b |
|  | 1/5 FD | 4.1 x 10^4^ b |
|  | FD | 3.5 x 10^4^ b |

^*^ The Kruskal-Wallis test was employed to test for significant differences in gene copy numbers followed by pairwise Wilcox test with FDR adjusted *P* value for multiple comparison.


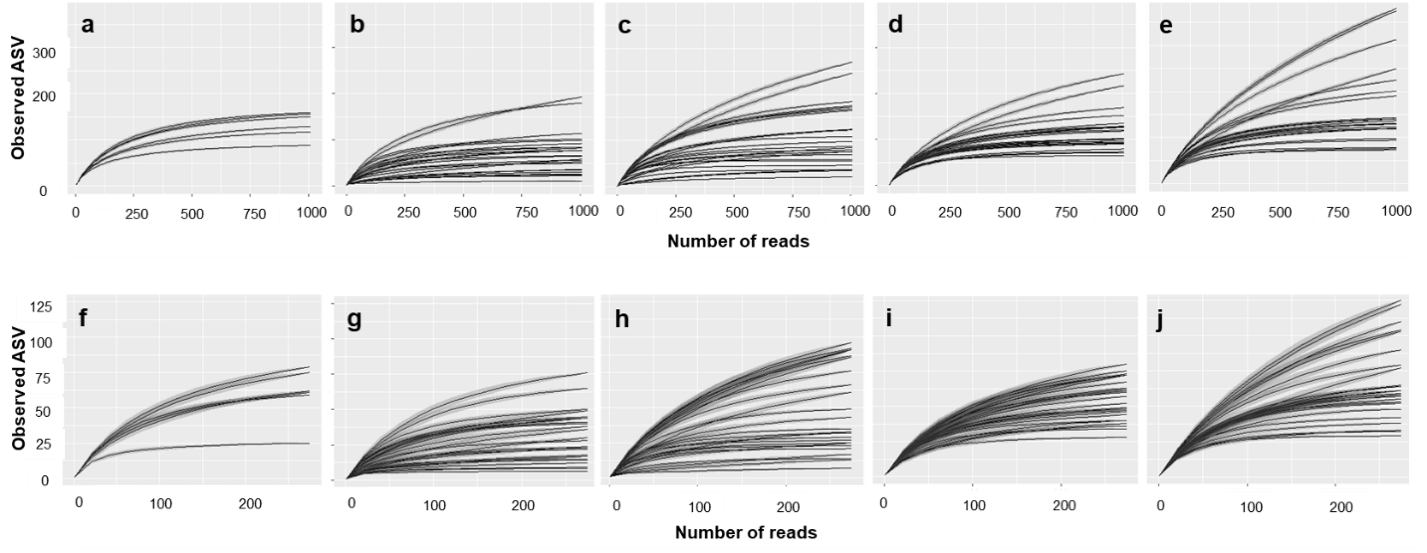


**Figure S1. Rarefaction curves showing the number of ASVs that were observed** **in lichens treated with different antimicrobial compounds.** Rarefaction curves are based on (a-e) the forward-read-only dataset and (f-j) the paired-end read dataset and derived from the (a,f) colistin (b,g), tetracycline (c,h), alkylpyrazine (d,i) and glyphosate (e,j) treatments.


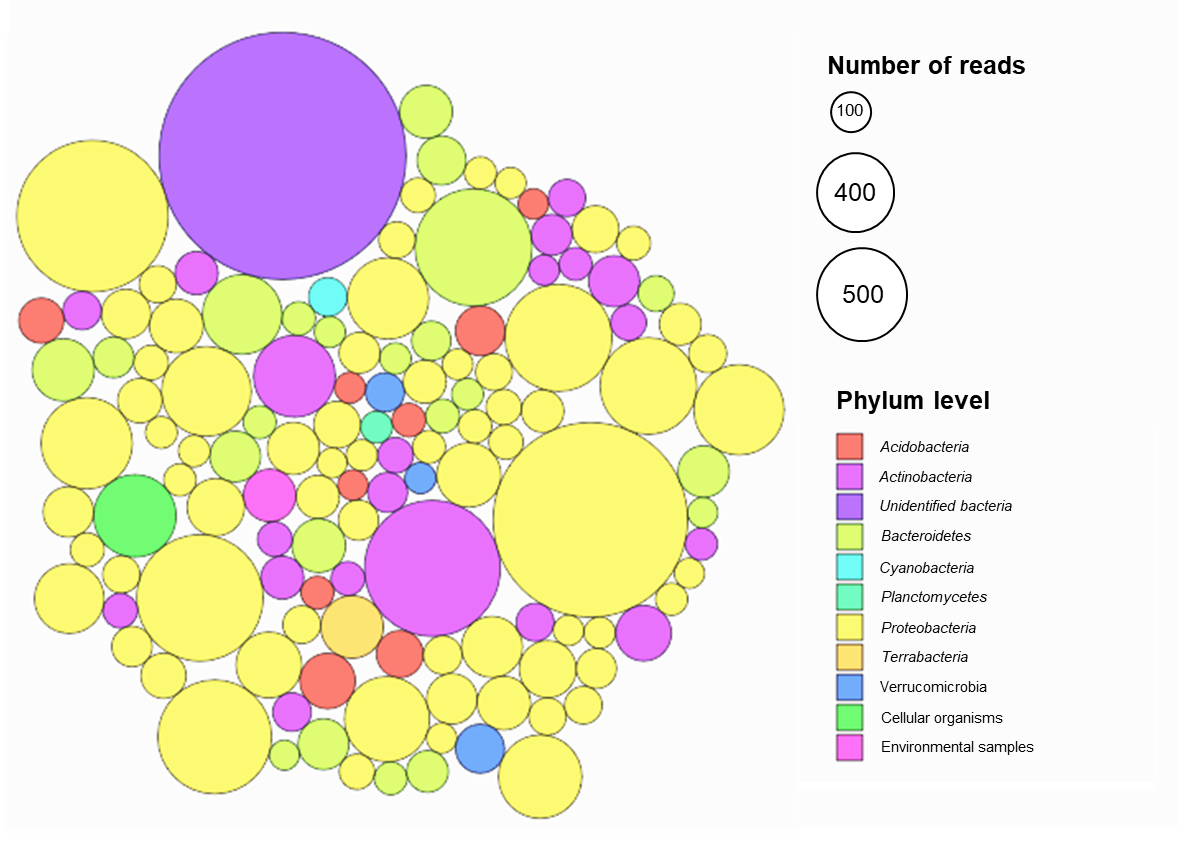


**Figure S2.** **Identification of carriers of the detected antimicrobial resistance genes**. The annotation was conducted using the metagenome classifier Kaiju and visualized with the integrated bubble plot tool.


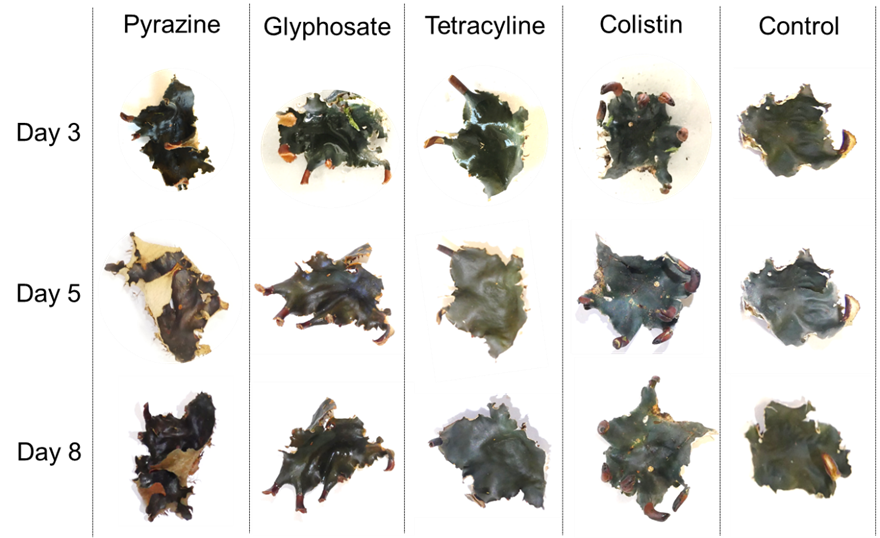


**Figure S3.** **Phenotypes of lichen samples that were treated with full dosages of antimicrobial compounds *i.e*. colistin, tetracycline, glyphosate, and alkylpyrazine in comparison to the untreated control.** Representative lichen samples were documented on day 3, 5 and 8 after the first spray application of the antimicrobial substances.


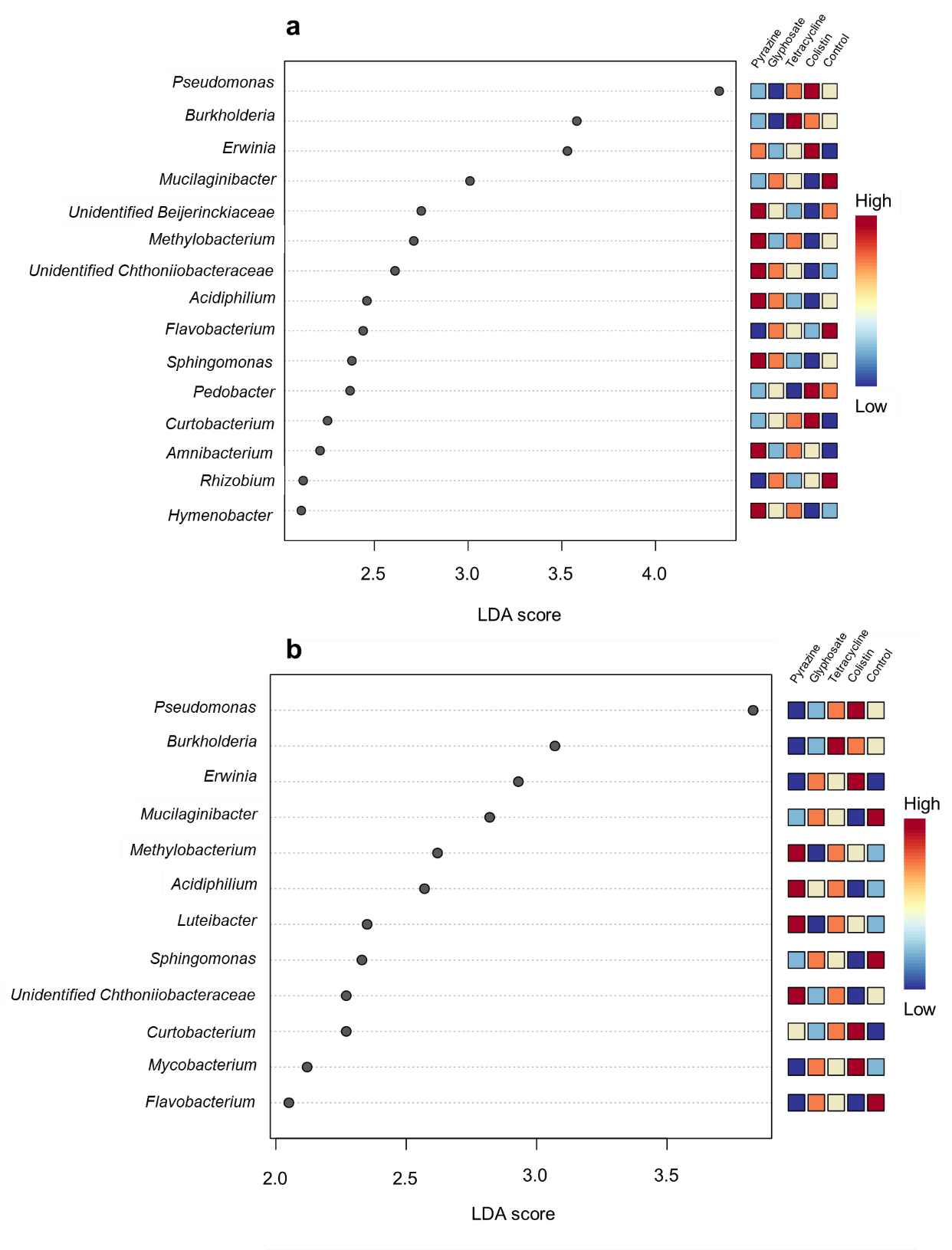


**Figure S4. Identification of responders to different antimicrobial compounds by LEfSe (Linear discriminant analysis effect size).** The analyses are based on (a) the total and (b) the active bacterial community. Only bacterial genera with a LDA score above 2 and cut-off *P* values below 0.05 were included**.**
